# Supplementary material for: The limits of action control for deceptive actions in sports: Response inhibition for the basketball pump fake
Source: PLoS One. 2025 Nov 13;20(11):e0332823. doi: 10.1371/journal.pone.0332823 (PMC12614604; doi:10.1371/journal.pone.0332823)
Supplement: S1 Appendix — (DOCX) [file pone.0332823.s001.docx]

**S1 Appendix**

**Further analysis of post-stop-trial adjustments**

All unsuccessful inhibitions (i.e., false responses as determined erroneous by buzzer release) can be further subdivided into erroneous initial responses and erroneous full responses. Erroneous full responses in 27.2%, and erroneous initial responses in 21.7% of all stop-trials (summing up to a total of 49.4% of erroneous responses in stop-trials). The remaining 0.03 % of erroneous responses were stop-trials, in which participants released the buzzer before the video started and were thus excluded from the analysis. The probability of successful and unsuccessful inhibitions in a stop-trial is shown in Figure S1, separated for erroneous initial responses and erroneous full responses. As the stop-signal interval approaches the point of ball release, participants are less able to suppress their responses, as can be seen. Consequently, the probability of releasing the table buzzer without jumping (erroneous initial response) decreases. Conversely, the probability of releasing the table buzzer and jumping up (erroneous full response) increases.

To further examine the strategic adjustments after a stop-trial, a repeated measures ANOVA was used with the additional subdivision of the unsuccessful stop-trials (for further details see results section) as the within subject-factor trial n-1 (go-trial, successful inhibition, erroneous initial response, and erroneous full response stop-trial). In addition, for one participant the erroneous full response condition were missing, thus the mean CE was used to fill the cell for the ANOVA calculation.

The mean CE in a go-trial after a go-trial was 84.5 ms (SEM = 7.06 ms), after a successful stop-trial 101.8 ms (SEM = 7.23 ms), after an erroneous initial response 119.3 ms (SEM = 8.85 ms), and after an erroneous full response 103.9 ms (SEM = 7.43 ms). A main effect was found for the factor trial n-1 [*F*(3,60) = 12.444, *p* < .001, *n_p_^2^* = .384]. The CE in a go-trial after a go-trial was smaller (i.e., higher precision) than after a successful stop-trial [*t*(20) = -3.684, *p* < .001, *d* = -804], after an erroneous initial response [*t*(20) = -5.427, *p* < .001, *d* = -1.184], and after an erroneous full response [*t*(20) = -4.848, *p* < .001, *d* = -1.058]. The response-precision performance after a successful stop-trial was better than after an erroneous initial response [*t*(20) = -2.797, *p* = .011, *d* = -.610]. The response-precision performance after a successful stop-trial and an erroneous full response was not significantly different (*p* = .659). There was no significant difference in response-precision performance after an erroneous full response and an erroneous initial response (*p* = .050).

The BRT in a go-trial after a go-trial was 223.3 ms (SEM = 10.04 ms), after a successful stop-trial 210.7 ms (SEM = 8.62 ms), after an erroneous initial response 193.9 ms (SEM = 12.05 ms), and after an erroneous full response 209.5 (SEM = 9.94 ms). For the BRT, the ANOVA showed a main effect for the factor trial n-1 [*F*(3,60) = 8.699, *p* < .001, *n_p_^2^ =* .303]. The BRT was significantly earlier (in relation to the target) in a go-trial after a go-trial than after a successful stop-trial [*t*(20) = 2.328, *p* = .031, *d* = .508], after an erroneous initial response [*t*(20) = 4.628, *p* < .001, *d* = 1.010], and after an erroneous full response [*t*(20) = 2.959, *p* = .008, *d* = .646]. The precision after a successful stop-trial was significantly earlier than after an erroneous initial response [*t*(20) = 2.709, *p* < .014, *d* = .591], but not after an erroneous full response (*p* = .793). The precision after an erroneous initial response was significantly later than after an erroneous full response [*t*(20) = -2.262, *p* = .035, *d* = -.494].

Participants left the ground (TOT) in a go-trial after a previous go-trial 63.1 ms (SEM = 9.69 ms) before the target, 42.5 ms (SEM = 8.51 ms) after a successful stop-trial, 28.1 ms (SEM = 9.67 ms) after an erroneous initial response, and 44.7 ms (SEM = 8.27 ms) after an erroneous full response. For the TOT, the ANOVA found a main effect for the within-subject factor trial n-1 [*F*(3,60) = 8.794, *p* < .001, *n_p_^2^* =.305]. The TOT was significantly earlier in a consecutive go-trial than after a successful stop-trial [*t*(20) = 3.764, *p* < .001, *d* = .821], as well after an erroneous initial response [*t*(20) = 4.854, *p* < .001, *d* = 1.059] and an erroneous full response [*t*(20) = 2.646, *p* = .015, *d* = .577]. The TOT after a successful stop-trial was significantly earlier than after an erroneous initial response [*t*(20) = 2.262, *p* = .035, *d* = .494]. The TOT after a successful stop-trial did not differ significantly from the TOT after an erroneous full response (*p =* .724). The TOT after an erroneous initial response was not significantly different than after an erroneous full response (*p* = .064). These results are in line with the response-precision performance.
