# Supplementary figures and images for: The limits of action control for deceptive actions in sports: Response inhibition for the basketball pump fake
Source: PLoS One. 2025 Nov 13;20(11):e0332823. doi: 10.1371/journal.pone.0332823 (PMC12614604; doi:10.1371/journal.pone.0332823)

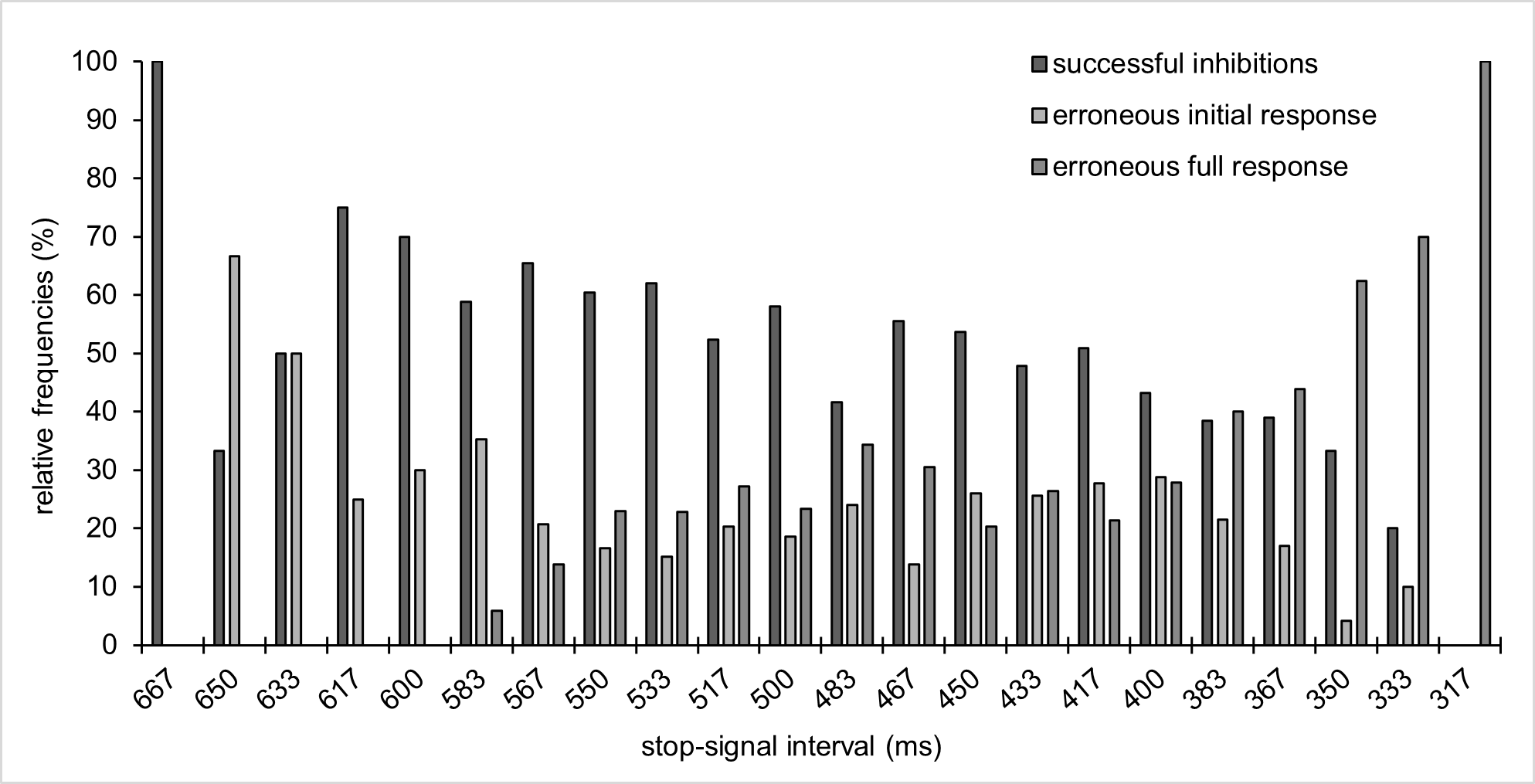

Supplement: S1 Fig — Frequencies (in percent) of the successful inhibitions (N = 633) and of the unsuccessful inhibitions differentiated for erroneous initial responses (N = 273), and erroneous full responses (N = 343), when responding to a pump fake across the different stop-signal intervals (in ms). (TIF) [file pone.0332823.s002.tif]
